# Negative predictive value and potential cost savings of acute nuclear myocardial perfusion imaging in low risk patients with suspected acute coronary syndrome: A prospective single blinded study

**DOI:** 10.1186/1471-227X-9-12

**Published:** 2009-06-19

**Authors:** Jakob L Forberg, Catarina E Hilmersson, Marcus Carlsson, Håkan Arheden, Jonas Björk, Krister Hjalte, Ulf Ekelund

**Affiliations:** 1 Divisions of Emergency Medicine, Department of Clinical Sciences, Lund University Hospital, Lund, Sweden; 2 Clinical Physiology, Department of Clinical Sciences, Lund University Hospital, Lund, Sweden; 3 Competence Center for Clinical Research, Lund University Hospital, Lund, Sweden; 4 Deptartment of Economics, Lund University, Lund, Sweden

## Abstract

**Background:**

Previous studies from the USA have shown that acute nuclear myocardial perfusion imaging (MPI) in low risk emergency department (ED) patients with suspected acute coronary syndrome (ACS) can be of clinical value. The aim of this study was to evaluate the utility and hospital economics of acute MPI in Swedish ED patients with suspected ACS.

**Methods:**

We included 40 patients (mean age 55 ± 2 years, 50% women) who were admitted from the ED at Lund University Hospital for chest pain suspicious of ACS, and who had a normal or non-ischemic ECG and no previous myocardial infarction. All patients underwent MPI from the ED, and the results were analyzed only after patient discharge. The current diagnostic practice of admitting the included patients for observation and further evaluation was compared to a theoretical "MPI strategy", where patients with a normal MPI test would have been discharged home from the ED.

**Results:**

Twenty-seven patients had normal MPI results, and none of them had ACS. MPI thus had a negative predictive value for ACS of 100%. With the MPI strategy, 2/3 of the patients would thus have been discharged from the ED, resulting in a reduction of total hospital cost by some 270 EUR and of bed occupancy by 0.8 days per investigated patient.

**Conclusion:**

Our findings in a Swedish ED support the results of larger American trials that acute MPI has the potential to safely reduce the number of admissions and decrease overall costs for low-risk ED patients with suspected ACS.

## Background

It has been estimated that some 180000 patients with chest pain suspicious of ACS (= acute myocardial infarction, AMI, or unstable angina pectoris) present at Swedish emergency departments (EDs) each year [1,2]. Current diagnostic methods for ACS in the ED, however, are clearly suboptimal. As a result, "rule-out" admissions are very common, and 7 out of 10 patients admitted for suspected ACS prove not to have it [1,3]. Also, many cases of ACS are diagnosed only after lengthy observation, with a resulting delay in therapy and an impaired prognosis. As many as 2–5% of those with ACS are erroneously sent home from the ED [4,5].

To overcome these problems, several new diagnostic methods have been suggested [6], e.g. echocardiography [7], multidetector CT scanning [8] and nuclear myocardial perfusion imaging (MPI) [9]. Acute MPI has been shown to be of value in routine care in the USA [10,11], primarily because of a high negative predictive value for ACS in patients with ongoing or recently abated chest pain and a non-diagnostic ECG. MPI may thus accurately identify patients who can be safely discharged directly from the ED. US studies also show that acute MPI can be cost effective[12]. To our knowledge however, no European study has yet evaluated the economy of acute MPI.

In the present study, the aim was to evaluate the utility and hospital economics of acute MPI in Swedish ED patients with suspected ACS.

## Methods

### Institution and patient material

Lund University Hospital (USiL) is a 1200 bed institution with fully public financing that serves a population of some 250 000, and has some 65 000 ED visits per year. Percutaneous coronary intervention (PCI) and coronary bypass surgery (CABG) are available 24 hours/day.

**Figure 1:**
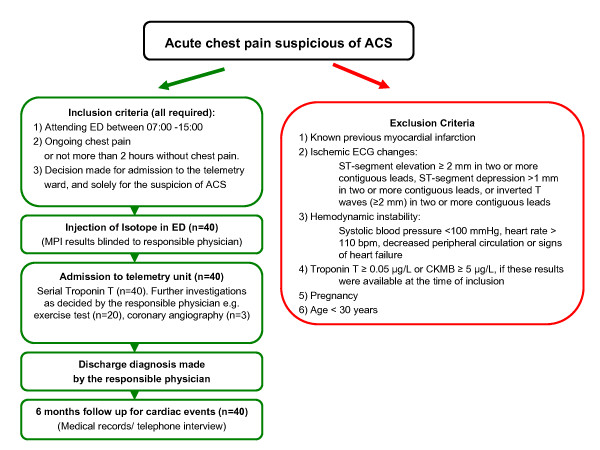
**Inclusion/exclusion criteria and diagnostic protocol**.

After informed consent, we prospectively included a convenience sample of 40 patients with chest pain suspicious of ACS attending the ED at USiL from 2002 to 2006. During the inclusion period, there was no systematic diagnostic protocol for patients with suspected ACS, no dedicated chest pain unit, and no formal strategy for admitting ED patients to in-hospital care. However, most admitted patients underwent serial blood testing and ECGs, as well as a pre-discharge exercise ECG when necessary. As far as known, no significant change in the usual care took place over the inclusion period. Inclusion and exclusion criteria are shown in figure 1.

Discharge diagnoses were made by the responsible physician according to European Society of Cardiology/American College of cardiology consensus documents using Troponin T as the critical biomarker [13], with a cut-off at 0.05 μg/L. In the study, diagnoses were noted "as is" from the patient records, and no further review was made. For patients with normal MPI results, the computerized patient records at USiL were used to identify ischemic cardiac events at 6 months after the index visit. Patients lacking notes of cardiac events at 6 months were interviewed by telephone to identify such events.

In January 2008, 1 EUR equalled 9.36 SEK. Unless stated otherwise, results are given as mean ± SEM.

### Myocardial scintigraphy

All patients underwent acute MPI after intravenous administration of a body-weight adjusted dose (7.5 MBq/kg) of 99mTc-tetrofosmin (MyoviewTM, GE Health care Life Sciences, Stockholm, Sweden) administered immediately after inclusion at the ED by personnel from the Division of Clinical Physiology. Electrocardiogram-gated (eight frames) single-photon emission computed tomography (SPECT) images were obtained in the supine position with a dual-head gamma camera (Vertex, ADAC Laboratories, Milpitas, CA, USA) according to a standardised clinical protocol. In short the acquisition was performed approximately 60–120 minutes after tracer injection, using a dual-head gamma camera with high-resolution, parallel-hole collimators. Data were collected at 32 projections over a 180 degree orbit, 40 seconds per projection, and 64 × 64 matrix zoomed to a pixel size of 5 mm. Attenuation correction was not used. SPECT images were reconstructed and post-filtered (Butterworth order, 5.0; cut-off fre quency, 0.6). The SPECT reconstruction and reorientation were automatically performed using Autospect plus (ADAC Laboratories), however, an experienced operator manually made corrections, if needed. The images were analysed in AutoQuant 4.3.1 (ADAC Laboratories). The images were interpreted by two physicians in consensus after patients were discharged from hospital and blinded to all clinical data and the discharge diagnosis. In this clinical setting a high sensitivity is required. Therefore, perfusion images had to be homogenous with no perfusion defects or attenuation artifacts, and EF and LV volumes had to be normal, in order for the scan to be read as normal.

### Economy

The hospital-related costs for each patient including all diagnostic procedures were retrieved from USiL's computerized economy system (PKS). All costs were the actual costs at the time of patient inclusion. The total cost of care and diagnostic work-up for each patient, excluding the cost for the study MPI, was considered to represent the cost of the current diagnostic practice. The cost of this approach was then compared to the cost of a theoretical management strategy where the same patient's MPI result was accessible to the ED physician and where the patient would have been discharged home from the ED directly after a non-pathological MPI result. For this theoretical strategy ("MPI strategy") the cost was postulated to be the total cost, including the cost of MPI, minus the cost of all in-hospital care and diagnostic tests performed after the ED. Length of stay and diagnosis at discharge were retrieved from USiL's computerized patient records systems (Melior, Siemens/PASIS, TietoEnator).

Since income data for the included patients were unavailable, the average cost of loss of societal production due to hospitalization was based on data on the mean salary for persons in Southern Sweden from the Swedish Central Bureau of Statistics, reflecting the expected background of the included patients. One day of lost production was assumed for patients admitted less than 24 hours. No cost for loss of production was calculated for patients over 65 years, as they were presumed to be retired.

### Ethical approval

The Research Ethics Committee at Lund University approved the study.

## Results

### MPI results and discharge diagnoses

The included 40 patients were on average 55 ± 2 years (35–80). Patients' characteristics are shown in Table 1. Twenty-five patients had ongoing chest pain at presentation and the remaining patients were without symptoms for an average of 1.1 hour. Twenty-seven patients had normal MPI results and none of these had ACS (Table 2). Sensitivity was thus 100% (2/2; 95% CI 16 – 100%) as was the negative predictive value (NPV). Specificity was 71% (27/38; 95% CI 54 – 85%) and the positive predictive value (PPV) was 15% (2/13). The false positive MPI results were found to be due to breast attenuation in five cases, inferior attenuation in three cases, inadequate quality of imaging in one case and two cases were classified as positive due to decreased left ventricular function only.

**Table 1:** Patient characteristics

	**Number (%)**
Men	20 (50)
Diabetes	2 (5)
Current smoker	11 (27)
Hypertension	9 (22)
Family history of ischemic heart disease	22 (55)
History of angina pectoris	10 (25)
History of CABG or PCI	2 (5)
Hypercholesterolemia	4 (10)
Congestive heart failure	0 (0)
History of stroke	3 (8)
Non-ischemic ECG changes	10 (25)

**Table 2:** MPI results and ACS outcome

	**MPI result**		***Total***
	Normal	Pathologic	
**Discharge diagnosis**			
No ACS	27	11	*38*
ACS	0	2	*2*

***Total***	*27*	*13*	*40*

Discharge diagnoses and length of stay for all 40 patients are shown in Table 3. The average length of hospital stay, for all patients and for patients with normal MPI, was 1.3 ± 0.2 days vs. 1.2 ± 0.2 days, respectively. At 6 months after the index visit, one of the 27 patients with normal MPI had been diagnosed with pericarditis (at one month), and none with ACS. A diagnostic strategy using acute MPI would thus potentially allow the immediate discharge of 67% (27/40) of the patients from the ED.

**Table 3:** Discharge diagnoses and length of stay for all patients

Number of patients (number of patients with normal MPI)						
	**Days in hospital**					
**Discharge diagnosis**	**<1**	**1**	**2**	**3**	**4**	**5**
Chest pain, unspecified	11 (7)	16 (12)	-	1 (1)	1 (1)	-
Supraventricular tachycardia (e.g atrial fibrillation)	-	3 (3)	-	-	-	-
Gastroesophageal reflux	1 (0)	1 (1)	-	-	-	-
Myalgia	-	2 (1)	-	-	-	-
Stable angina pectoris	-	1 (1)	1 (0)	-	-	-
Acute subendocardial infarction	-	-	-	-	1 (0)	1 (0)

*Number of patients*	*12 (7)*	*23 (18)*	*1 (0)*	*1 (1)*	*2 (1)*	*1 (0)*

### Economics of the current diagnostic practice versus an MPI strategy

Diagnostic tests and related costs for all 40 patients are presented in Table 4. Length of stay accounted for 71% of the total admission cost with the current diagnostic practice. Table 5 shows total hospital costs for the current diagnostic practice and the MPI strategy. As can be seen, the MPI strategy would potentially reduce costs by 267 ± 96 EUR and bed occupancy by 0.8 ± 0.16 days per patient undergoing MPI.

**Table 4:** Diagnostic tests after the ED and related costs for all patients

	**Cost **(EUR)
**Length of stay **(n = 40)	745 ± 81
**Diagnostic tests**	
Blood samples	24 ± 3
Exercise-ECG (n = 15)	118 ± 1
X-ray (n = 12)	98 ± 37
Coronary angiography (n = 3)	730 ± 166
Echocardiography (n = 3)	120 ± 6
Myocardial Perfusion Imaging	283 ± 8
***Total in-ward cost ****(n = 40)*	895 ± 120
***ED cost (excl. MPI) ****n = 40*	344 ± 10

**Table 5:** Costs and potential reduction of costs with MPI strategy

	**Pr Patient** EUR
**Cost according to diagnostic strategy**	
Current diagnostic approach	1239 ± 119
MPI strategy	973 ± 118
**Consequences of using MPI strategy**	
Reduction of costs	266 ± 96
Reduction of days in hospital	0.8 ± 0.16 Days

Excluding patients >65 years, there were a total of 18 potentially saved work days (144 h), corresponding to a reduction in production loss of 69 EUR per patient undergoing MPI.

## Discussion

Our results suggest that acute MPI in selected low risk ED patients with suspected ACS can safely reduce hospital admissions by some 2/3. Such a reduction would result in a saving of about 0.8 bed days and 270 EUR per patient investigated with MPI.

Our findings support previous results from larger North American trials that acute MPI can reduce costs and admittance to in-hospital care in low or moderate risk patients with suspected ACS, with preserved diagnostic safety. In a large multi-center randomized trial, Udelson et al[10] found that in patients with chest pain and a non-ischemic ECG, MPI reduced unnecessary hospitalizations by 20%. In a smaller randomized study [14] with similar patients, an acute MPI strategy significantly reduced the median length of hospitalization by 2 days and the overall median cost by 49%.

Although the conclusions from our study should be limited due to the small sample size, our results indicate that these North American findings are also valid in a Scandinavian setting. In previous small European trials [15,16], MPI was found to have a negative predictive value for ACS of 96%. These results are however difficult to compare with ours, since a delay of up to six hours after symptom presentation to isotope injection was allowed. Other studies [9] have shown that the sensitivity and negative predictive value declines with injections later than 2 hours after symptoms.

So far only two studies [16,17] have analyzed MPI performance with the newer AMI definition using troponin as a biomarker, as in the present investigation. In one of the studies [17], the sensitivity of acute MPI was only 75% when performed in patients with a moderate (instead of low) ACS risk, which in spite of an openly negative MPI still were admitted for serial biomarker sampling. Due to the unacceptable low sensitivity the authors concluded that MPI as a diagnostic tool was suboptimal in patients with a moderate risk of ACS. It thus seems as acute MPI should only be used to reduce "unnecessary" admissions in low risk ACS patients, and that the individual chest pain patient should be thoroughly risk-stratified before deciding the diagnostic method.

Because of the ability to select patients for ED discharge, MPI has been jointly recommended by the American College of Cardiology/American Heart Association/American Society of Nuclear Cardiology [18] in low risk ED patients with suspected ACS and a non-ischemic ECG. An additional advantage with MPI is that it is well suited for telemedicine applications, reducing the need for on-site physicians for MPI interpretation. Since the perfusion is imaged, there is also a potential for earlier detection of ACS, but published positive predictive values are low [10,19]. In the present study, the PPV was only 15%, which indicates that the clinical value of MPI for this purpose is very limited.

The benefits of introducing MPI of course depend on the local standard of care. In the present study, the average 1.3 days of hospital stay could have been reduced by 0.8 days with the MPI strategy. In comparison, a mean length of stay of 3.8 days in another study [14] was cut to 1.4 days when MPI results were available to the ED physicians, with no change in patient outcome.

Disadvantages with MPI include that the test itself is expensive, and that it exposes the patient to radiation, in our case approximately 3 mSv. In the present study, personnel from the department of Clinical Physiology brought the isotope to the ED and injected it into the patient. If this is not practical, implementation of MPI in routine care will likely require training of ED personnel, adoption of guidelines for handling isotopes, and perhaps even rebuilding rooms for radiation safety. MPI would probably not be suitable for centers where nuclear cardiology experts are not present or where the patient volume is small. With an annual attendance at our ED of some 65000 patients, we predict that there will be one or two patients per 24 h suitable for acute MPI. Due to the relatively high cost of the MPI itself, it seems important to ascertain that only patients who would otherwise be admitted to in-hospital care are referred to MPI. If not, as with any new diagnostic test, there is a risk of overuse which would decrease the potential cost savings. Another risk is that false positive MPI results induce unnecessary and expensive further testing, which will also reduce cost savings. When implementing MPI in routine care, it seems essential to inform the physicians about the very low PPV in these patients.

Several other new diagnostic methods have been suggested to be of value in the chest pain patient with suspected ACS [6]. Coronary angiography using multidetector CT scanning (MDCT) has shown promising results and in a meta-analysis by Vanhoenacker et al. the pooled sensitivity and specificity were 95% and 90% [20] in detecting non-ST-elevation ACS. MDCT has the advantage over MPI to be a very rapid investigation and to be available in more centers and more often outside office hours. MDCT also has the potential to detect other causes of chest pain than acute cardiac disease. A disadvantage with MDCT is that it exposes the patient to a larger radiation dose (5–20 mSv) than rest MPI.

Extending MPI availability outside office hours would most likely increase the cost per MPI investigation. The exact cost increase will of course be different at every center, but a larger patient volume than ours would probably be needed to make an on-call physician and standby isotope economically feasible. In our hospital, about one patient a day during office hours can be acutely imaged within the existing capacity of the MPI-cameras.

### Limitations

Our study only included a small fraction of the potentially eligible subjects during the study period, which in theory could lead to a selection bias. There were however no systematic criteria for patient selection other than the inclusion criteria described in Methods, and the included patients were therefore considered to be a random sample of all eligible patients. The patients included in this study were on average eight years younger than our chest pain patients in general [21]. This probably reflects our exclusion criteria (e.g. normal ECG and no previous AMI) but could of course also be caused by a general tendency to include younger patients, which would then in turn lead to an overestimation of the working days saved using the MPI strategy. The prevalence of ACS was low in our study, and this could theoretically reflect a selection of patients with a particularly low risk of ACS, actually suitable for ED discharge without an MPI. However, our ACS prevalence was comparable to that in previous studies [10,19], and the hospital admission of our patients was decided before inclusion in the study. Changes in the cost of care of course occurred during the inclusion period, but these changes were small. If anything, the cost changes would cause us to underestimate the cost reduction with a future MPI implementation. There was no review of the patients' discharge diagnoses. On the other hand, the follow-up revealed that none of the patients with normal MPI results had had an ACS at 6 months.

This study only evaluated MPI as a means to identify patients suitable for discharge home from the ED, i.e. to exclude ACS. Underlying coronary artery disease could still exist in the patients with a negative MPI, at least in the 12 patients who were not evaluated with exercise ECGs or coronary angiography during the 6 month follow up period. We consider it unlikely however, since in these cases the attending physician did not consider further investigations necessary to exclude coronary artery disease.

## Conclusion

For ED patients with suspected ACS, a normal or non-ischemic ECG and no previous AMI, this study confirms that acute MPI has the potential to safely reduce admissions to in-hospital care as well as costs. This would allow limited health care resources to be focused on patients with true ACS, where rapid intervention clearly improves the prognosis.

## Competing interests

The authors declare that they have no competing interests.

## Authors' contributions

JLF participated in the design of the study, data acquisition, data analysis, and wrote the manuscript. CEH collected and analysed the economical data and wrote the manuscript. JB participated in the statistical analysis and in the critical revision of the manuscript. MC and AH made the MPI interpretations and made critical revisions of the manuscript. KH participated in the economical analysis. UE participated in the conception and design of the study, data analysis, managed the project and wrote the manuscript. All authors have read and approved the final version of the manuscript

## Pre-publication history

The pre-publication history for this paper can be accessed here:


